# Oxygen intercalation in PVD graphene grown on copper substrates: A decoupling approach

**DOI:** 10.1016/j.apsusc.2020.147100

**Published:** 2020-07-11

**Authors:** J. Azpeitia, I. Palacio, J.I. Martínez, I. Muñoz-Ochando, K. Lauwaet, F.J. Mompean, G.J. Ellis, M. García-Hernández, J.A. Martín-Gago, C. Munuera, M.F. López

**Affiliations:** a Materials Science Factory, Instituto de Ciencia de Materiales de Madrid, Consejo Superior de Investigaciones Científicas, Cantoblanco ES-28049, Madrid, Spain; b Instituto de Ciencia y Tecnología de Polímeros, Consejo Superior de Investigaciones Científicas, ES-28006 Madrid, Spain

**Keywords:** Graphene, Intercalation, XPS, AFM, LEED

## Abstract

We investigate the intercalation process of oxygen in-between a PVD-grown graphene layer and different copper substrates as a methodology for reducing the substrate-layer interaction. This growth method leads to an extended defect-free graphene layer that strongly couples with the substrate. We have found, by means of X-ray photoelectron spectroscopy, that after oxygen exposure at different temperatures, ranging from 280 °C to 550 °C, oxygen intercalates at the interface of graphene grown on Cu foil at an optimal temperature of 500 °C. The low energy electron diffraction technique confirms the adsorption of an atomic oxygen adlayer on top of the Cu surface and below graphene after oxygen exposure at elevated temperature, but no oxidation of the substrate is induced. The emergence of the 2D Raman peak, quenched by the large interaction with the substrate, reveals that the intercalation process induces a structural undoing. As suggested by atomic force microscopy, the oxygen intercalation does not change significantly the surface morphology. Moreover, theoretical simulations provide further insights into the electronic and structural undoing process. This protocol opens the door to an efficient methodology to weaken the graphene-substrate interaction for a more efficient transfer to arbitrary surfaces.

## 1 Introduction

Over last decade, since the discovery of graphene [1], many methods have been developed for its synthesis and among them chemical vapor deposition (CVD) is the most widely employed [2]. A variant of this technique where a solid source of carbon is used, physical vapor deposition (PVD), is one of the most appealing protocols [3,4] as it lowers the temperature required during the growth process and results in layers with a low number of structural and chemical defects. Amongst the growth methods that employ solid carbon sources, a novel method using C_60_ as precursor molecules under ultra-high vacuum (UHV) conditions has been recently successfully demonstrated. Although proven to be an effective process to obtain complete, high-quality monolayer graphene over polycrystalline copper foils [2,4], the coupling of the graphene layer with the copper is significant. Presently, metal substrates are incompatible with the exploitation of the unique electronic properties of graphene, making transfer protocols to more technological substrates mandatory. The problems that can emerge upon transfer that are derived from the interaction with the substrate can be avoided if the interactions can be deactivated or, at least, diminished.

One of the most promising protocols for the transfer of metal-grown graphene is the so-called electrochemical delamination method [5], a simple chemical process that has been demonstrated to be very effective and allows substrate recycling. The procedure consists in the use of the graphene/metal system as one of the electrodes (cathode, negatively biased) for a typical electrolysis process. The protons, released from the electrochemical reaction, intercalate through graphene. This method has been widely used on systems with a copper substrate, due to its relatively low interaction with the graphene layer. However, due to the perfection of the layer, PVD grown graphene shows a strong coupling with the substrate, and the strategies developed for the CVD layers fail when applied to it. In order to be able to transfer the graphene layer via the delamination method in systems where the interaction between the graphene layer and the substrate is important, this interaction must be suppressed. In this respect, there have been many reports on the graphene decoupling from strongly interacting substrates such as iridium, ruthenium or rhodium, amongst others [6–9]. In those cases, the method used to decouple the graphene is intercalation. Different chemical elements have been employed for this purpose, such as hydrogen, oxygen, gold or cesium [10–13], with different substrates, and by applying different techniques, such as intercalation in UHV, electrochemical intercalation, photo-dissociation-mediated intercalation, etc. [14–16]. In fact, from results obtained by intercalating different elements underneath the graphene layer it is possible to tune the electronic properties of the system, obtaining different graphene materials ranging from a strong n-type doping to a strong p-type doping [7].

Oxygen intercalation has been previously studied on many different metal surfaces, such as Gr/Ru(0001) [11,14], Gr/Ir(111) [8], Gr/Pt [17,18] and Gr/Rh [19] systems. In the specific case of graphene on Cu substrates [20–31], most of the previous studies focus on describing the oxidation process mainly aiming at determining whether the graphene layer guarantees protection against oxidation/corrosion for Cu substrates [20–26]. In these works, the substrate under the graphene layer becomes oxidized by exposing the sample to (near) ambient conditions or by using a water-assisted atmosphere. In addition, some studies deal with the decoupling of the graphene from the substrate to facilitate the transfer process [20,27,28]. However, the intercalation process by controlled exposure to a pure oxygen atmosphere under UHV conditions has not yet been explored. All of the previous reports on Cu substrates have in common the use of CVD graphene. As already mentioned, the PVD graphene grown using C_60_ as a precursor is defect-free and therefore it highly interacts with the Cu substrate [4]. We show that a controlled oxygen intercalation process structurally and electronically decouples this highly linked interface, making this process favourable as a first stage for transferring graphene onto other surfaces.

In this work, we report on the oxygen intercalation process applied to PVD grown graphene on polycrystalline Cu substrates. The process has been carried out in a UHV environment in order to have a controlled and pure oxygen exposure, that allows fine tuning and optimization of the different parameters (temperature, coverage, etc.) The process has been studied in detail by means of Low Energy Electron Diffraction (LEED), X-Ray Photoelectron Spectroscopy (XPS) and Raman Spectroscopy. In addition, Atomic Force Microscopy (AFM) and Kelvin Probe Force Spectroscopy (KPFS) have been used for topographic and surface potential measurements, respectively.

## 2 Experimental

Graphene samples were grown in a UHV environment with a base pressure of 1 × 10^-10^ mbar on different types of Cu substrates, both polycrystalline and single crystals. Commercial 35 μm thick Cu foils were used as polycrystalline substrates. In order to observe differences between the as-grown and the oxygen-intercalated specimens after graphene growth, samples were cut into two pieces (twin samples). One was oxygen-intercalated while the other was used as a reference. Both were characterized by means of the aforementioned techniques. In addition to copper foils, single crystals of Cu(100) and Cu(111) were initially employed to assess the feasibility of the intercalation method with a more controlled system. The surfaces of these substrates were prepared by following a standard cleaning procedure in UHV consisting of successive cycles of argon sputtering and annealing at 800 °C. The substrate temperature was monitored with an optical pyrometer. Commercial fullerene C_60_ molecules (sigma Aldrich 98% purity) were used as the precursor species for graphene growth on the substrate. The fullerenes were purified for several hours at 500 °C. For graphene growth, the clean Cu substrate was heated to 800 °C and maintained at this temperature. Then, the C_60_ molecules were sublimated in UHV at a temperature of 450 °C and deposited onto the hot surface during typically 1 h [4].

The oxygen intercalation procedure was carried out in UHV by controlling the incoming oxygen (99.9999% purity) dose with a leak valve. The temperature of intercalation was optimized by testing various samples at different intercalation temperatures of 280 °C, 350 °C, 400 °C, 500 °C and 550 °C, monitored using a K-type thermocouple attached to the heater. In all cases the oxygen dose was set to 20×10^3^ L (Langmuir, where 1L = 10^-6^ Torr s), using an oxygen pressure of 1.1×10^-5^ mbar for the intercalation procedure during 30 min. In order to avoid oxygen desorption from the samples, after completing the intercalation procedure, the oxygen pressure was maintained until the sample was cooled to RT.

After oxygen intercalation, an exhaustive characterization was undertaken in order to elucidate the mechanism of the process. The samples were characterized *in situ* with an OMICRON LEED. Subsequently, they were transferred in air to a different UHV chamber, where oxygen intercalation and XPS characterization were carried out. Photoemission experiments were performed with a hemispherical analyzer SPECS Phoiboss 100 MCD-5 with an instrumental resolution of 0.02 eV. Al K_α_ radiation (1486.7 eV) was employed as X-ray source. The topographic characterization of the samples prior to and after oxygen exposure was performed ex situ with a commercial AFM from Nanotec. The images were acquired in dynamic mode to preserve the integrity of both the samples and the tip. Using the same equipment, Kelvin Probe Force Spectroscopy (KPFS) measurements were performed to determine the surface potential. The tips employed were specially manufactured for KPFS measurements coated with gold nanoparticles (2–3 nm) from Nextip [32]. HOPG was used as a reference sample. All images and surface potential curves were analyzed with WSxM software [33]. Raman measurements were performed with a Renishaw InVia Reflex equipment using an argon ion 514.5 nm (2.41 eV) laser with a laser power at the sample < 2 mW, a 100x (NA 0.85) objective and a Peltier-cooled CCD detector.

Density Functional Theory (DFT)-based calculations were performed by using the plane-wave code QUANTUM ESPRESSO [34], including the semiempirical vdW DFT + D3 correction [35] to add dispersive forces. For this purpose, the revised version of the generalized gradient corrected approximation of Perdew, Burke, and Ernzerhof (rPBE) was used to account for the exchange–correlation effects (XC) [36]. Rabe-Rappe-Kaxiras-Joannopoulos (RRKJ) ultrasoft pseudopotentials [37] were used to model the ion–electron interaction in the C, O and Cu atoms. The Brillouin zones (BZ) were sampled by means of: i) [4 × 8 × 1] Monkhorst-Pack grids [38] for the structural and lattice optimizations, and ii) [32 × 64 × 1] Monkhorst-Pack grids for the electronic structure calculations. To guarantee a full convergence in total energy and density, the one-electron wave-functions were expanded in a basis of plane-waves with energy cut-offs of 40 and 300 Ry for the kinetic energy and for the electronic density, respectively. Simultaneously to the structural relaxations, full cell-shape relaxations were carried out with two different algorithms: i) a damped dynamics, checking both Parrinello-Rahman [39] and Wentzcovitch [40] implementations, and ii) a bfgs-like relaxation. The different approaches provide similar cell-shapes, lattice parameters and frequencies for the internal vibrations and cell oscillations. This simultaneous lattice and structural relaxation provides an optimal strain minimization up to achieve a global stress < 0.05 GPa in both the metal substrate and the graphene lattices. A description of the geometrical model is explained in detail in the supplementary information (Fig. S6).

## 3 Results

**Fig. 1:**
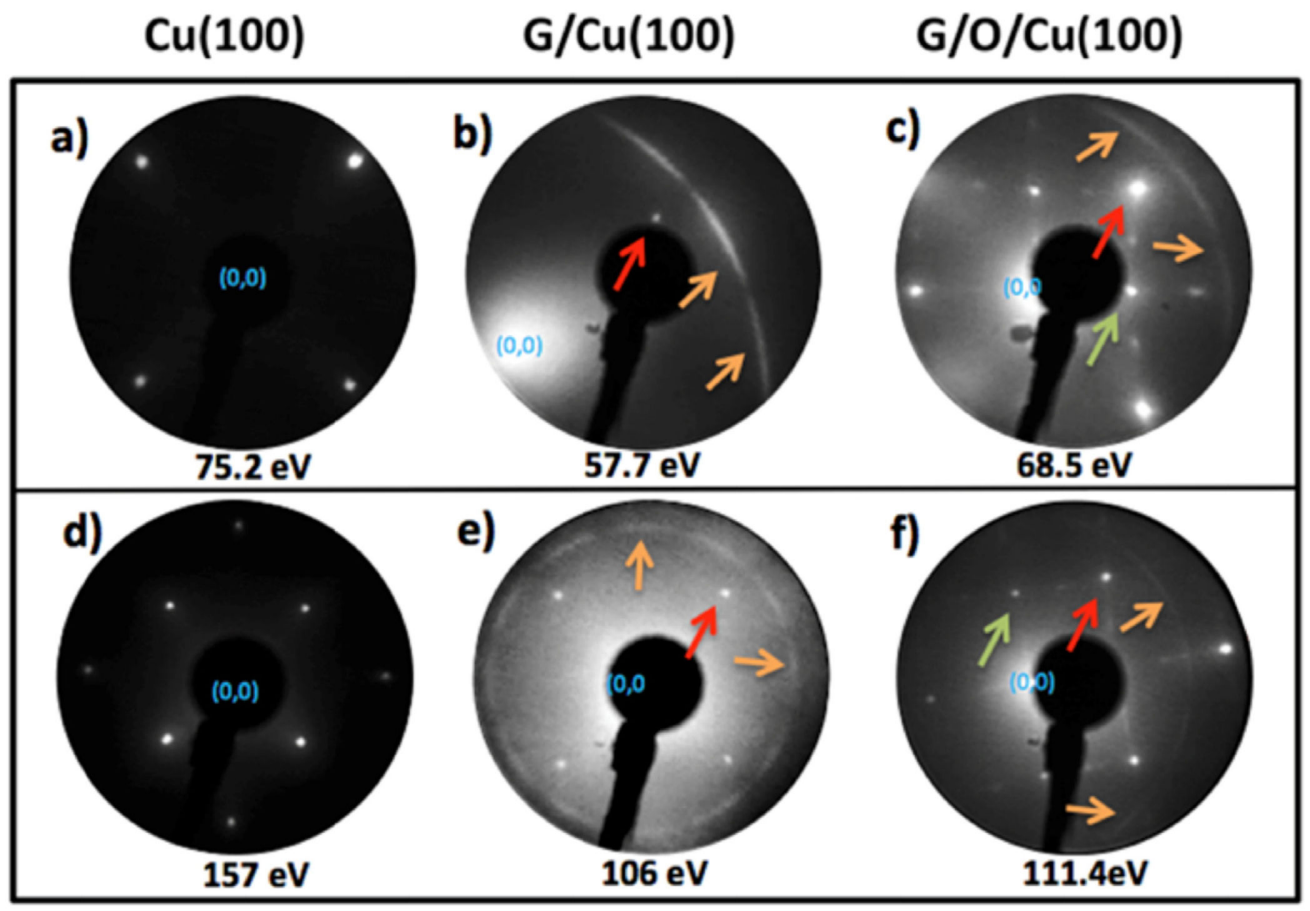
LEED patterns acquired at different energies of G on Cu(100). The red, green and orange arrows indicate the copper, the oxygen ($\sqrt{2}$ × $\sqrt{2}$)R45° reconstruction and the graphene signals, respectively. The first column (a and d) corresponds to bare Cu(100) surface, the second (b and e) to the graphene on top of the copper and the third one (c and f) to the oxygen intercalated between the graphene and the copper.

The main objective of this work is to study the oxygen intercalation process in the system formed by PVD graphene on Cu foil samples. With this aim, it was first necessary to determine the best conditions for the intercalation process, which are defined by the temperature range and the minimum oxygen dose. To address this problem, a fast and reliable feedback based on LEED patterns was used. As in polycrystalline samples, grains with different orientation contribute with different spots to the LEED pattern without possibility of precise identification [4], the use of single crystals was mandatory. For this reason, we have studied PVD graphene grown on two differently oriented single crystals: Cu (111) and Cu(100), as representative of the polycrystalline structure of the Cu foil. Oxygen on Cu(100) has been extensively studied and an oxygen adsorbed overlayer was widely reported [41,42]. Fig. 1a and 1d show representative LEED patterns at different energies for the Cu (100) surface, where bright spots with a very low background can be observed, confirming the cleanness of the substrate prior to the growth. LEED patterns recorded after the graphene growth (Fig. 1b,e) show the emergence of a ring, which corresponds to the polycrystalline growth of graphene. The red arrows indicate some of the spots corresponding to Cu, while the orange arrows indicate the graphene ring. Fig. 1b has been recorded at low energy and out-of-normal to enhance the signal on the graphene ring. In this figure it is observed that the ring shows some modulation, providing evidence for a not fully random orientation of the graphene domains [43]. A similar graphene ring was observed when using the Cu(111) single crystal as substrate (see Fig. S1).

The subsequent intercalation of oxygen on the Cu(100) single crystal was also monitored by LEED. Thus, after graphene growth the sample was annealed at 400 °C while being dosed with 20×10^3^ L of oxygen, and Fig. 1c and 1f show the LEED patterns obtained after exposure. In addition to the spots corresponding to Cu(1 00) (red arrows) and the graphene ring (orange arrows), both LEED patterns manifest the appearance of new spots (some marked with green arrows) not observed prior to oxygen exposure. They correspond to a ($\sqrt{2}$ × 2$\sqrt{2}$)*R*45°surface reconstruction, exhibiting two different domains. As it is already known, oxygen exposure on Cu(100) at room temperature and low coverage induces the formation of a c(2×2) superstructure, while at high temperatures the surface reconstructs forming a ($\sqrt{2}$ × 2$\sqrt{2}$)*R*45° surface structure [41,42]. This latter reconstruction is referred as a missing row structure due to the ejection of every fourth atomic surface row of Cu atoms in the [010] direction [41]. The observation, after an oxygen dose at 400 °C, of both the (2 × 22)*R*45° reconstruction and the graphene ring in the LEED pattern indicates that the oxygen has been able to induce the reconstruction of Cu beneath the graphene surface. The intensity of the spots corresponding to the oxygen superstructure is constant all along the surface, indicating that the intercalation of oxygen is uniform in the sub-mm scale (electron beam size). Nonetheless, for smaller length scales oxygen differences at the graphene/Cu interface could be found. Additionally, the reproducibility of the KPFS data (Fig. S4) along the surface of the samples supports the uniformity of the intercalation process within the micrometric scale.

**Fig. 2:**
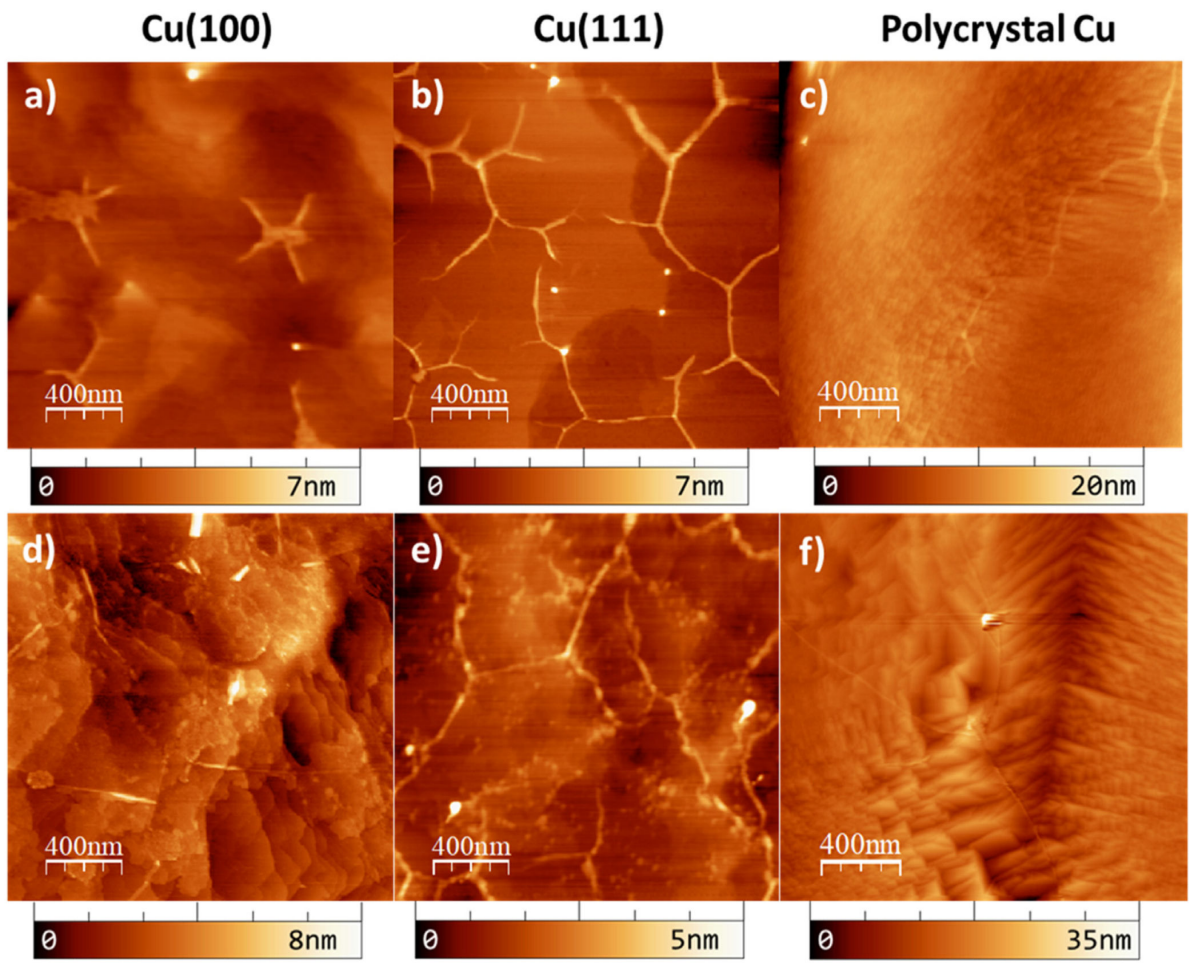
AFM topography images acquired in dynamic mode. Top panel shows the graphene layer on top of different Cu substrates: a) Cu(100) single crystal, b) Cu (111) single crystal and c) polycrystalline Cu foil. Bottom panel shows the surface after the oxygen intercalation process, for the same surface orientations as shown in the top panel. d) Cu(100), e) Cu(111) and f) polycrystalline Cu foil.

To verify the surface morphology, the graphene/Cu samples were characterized by AFM prior to and after oxygen intercalation for the Cu (100) and Cu(111) single crystal substrates and the polycrystalline Cu foils. The top panel of Fig. 2 summarizes the AFM topographic measurements prior to intercalation process. While the single crystals (Fig. 2a and b) show flat surfaces with well-defined large terraces in the order of microns, the polycrystalline substrate (Fig. 2c) exhibits a higher density of step edges, with an increase in the overall roughness. The bottom panel in Fig. 2 presents the AFM measurements after oxygen intercalation. Although changes are visible in the morphology of the substrate surface, with alterations in the shape of the copper terraces, graphene wrinkles remain visible and unaltered, clearly suggesting the preservation of the graphene layer on top of the intercalated oxygen. AFM images of the different stages of the graphene growth and oxygen intercalation processes for the Gr/Cu(111) sample are shown in Fig. S3. It is important to remember that the graphene coverage is one complete monolayer, as the deposition PVD method [4] is a self-limiting process, and the random areas checked by AFM show indeed a complete coverage.

Both, the presence of the wrinkles and the ring observed by the LEED patterns indicate that the graphene layer grown on the Cu substrates is polycrystalline (see Fig. 1, Fig. S1 and ref. 4 for Cu(100), Cu (111) and Cu foil, respectively). Therefore, graphene grain boundaries could be the channels for oxygen intercalation, as supported previously [44].

Once the feasibility of the intercalation method is proved (oxygen is indeed intercalated between graphene and copper and no damage is observed on the graphene layer for the dose employed) we focus on the PVD graphene on Cu foil, since this system is the most interesting for future applications. To understand the chemical processes involved in the intercalation approach and for a further optimization of the intercalation conditions, we performed XPS in the regions corresponding to the C 1s, O 1s and Cu 2p emissions. The graphene grown on Cu foil was exposed to the same oxygen dose (20×10^3^ L) at different temperatures. A temperature increase should enhance oxygen diffusion, and hence promote the intercalation process, although it is important to consider that an excessive temperature could compromise the graphene layer through etching processes. The XPS Cu 2p spectra with exposure temperatures from 280 °C to 550 °C are shown in Fig. S2.

**Fig. 3:**
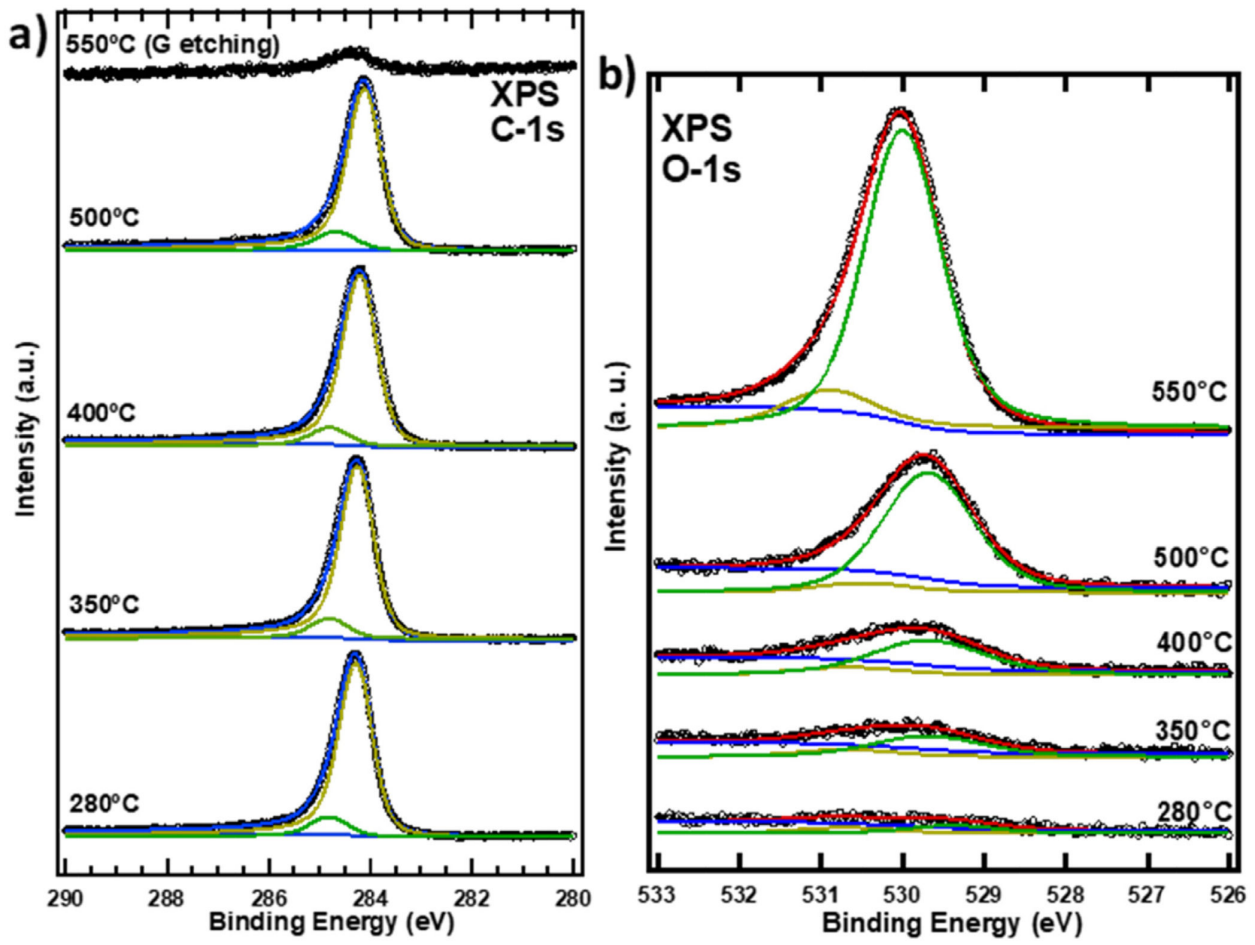
Evolution of the XPS spectra of graphene grown on polycrystalline Cu foil with the temperature after oxygen exposition (20×10^3^ L) for: a) C1s core level and b) O1s core level.

Fig. 3a shows the XPS C1s spectra as a function of the temperature upon oxygen exposure. The C 1 s peak located at 284.21 eV, corresponding to the sp^2^ emission, was fitted using a Doniach and Sunjic function with an asymmetry parameter of 0.068 [45]. A second peak located at 284.81 eV was required for optimal fitting of the experimental data and a Voigt curve was used in this case. This small peak is present from the start of the annealing process and it is ascribed to sp^3^ carbon [15,46], which mainly comes from the graphene grain boundaries. The contribution of sp^3^ carbon, as already observed in previous works, can be low and even almost negligible [25]. As it is observed in Fig. 3a, there is no so much difference between the XPS C1s spectra from 280 to 500 °C as no new components appear during the intercalation process. This indicates a lack of chemical interaction between the graphene layer and the intercalated oxygen. The only change observed when increasing the temperature is related to the position of the sp^2^ peak, where a slight down shift of 0.08 eV for 400 °C and 0.13 eV for 500 °C. It must be noticed that prior to the intercalation process, our graphene layer shows a non-negligible interaction with the substrate that leads to a n-doping behaviour, as previously reported [4]. The down shift in binding energy (BE) observed during this intercalating process indicates that the initial n-doping of the as-grown graphene layer diminishes upon intercalation, as described by Michely et al. [47]. This decrease of the n-doping character of the graphene layer reflects an un-doping effect with respect to the as-grown layer, evidencing that the underlying oxygen atoms decouple the graphene layer. Graphene n-doping was previously observed in graphene on iridium [7,47] as well as on Cu foils [23,24,48], although in our system it is lower. However, it should be noted that for the graphene on iridium, the interaction between iridium and graphene prior to intercalation is stronger than in the case of Cu. For this reason, different binding energy values corresponding to the sp^2^ emission would be expected for the different systems. Moreover in all the previous studies, the graphene was grown by CVD in contrast to our PVD method, and the oxygen intercalation process was also different (ambient air exposure) in contrast to our procedure based on controlled exposure to pure oxygen. Both differences lead to different doping and un-doping levels of the graphene layer for the as-grown and oxygen intercalated layer, respectively. Interestingly, a previous work in graphene on Cu single crystals reported that the electronic response after oxygen intercalation is different for different orientations of Cu [49]. Therefore, the results presented in this work would reflect an average response over the different orientations present in the Cu foil substrate. Finally, we found values of around 550 °C for the lowest onset temperature for etching 1 ML graphene, higher than those in any other reported systems [23,50]. It has already been reported that the etching process of graphene with oxygen or hydrogen starts at the defects of the graphene layer [51], and that the presence of a partial graphene layer diminishes the etching temperatures values [50]. In our case, the fact that the etching process occurs at a higher temperature is consistent with a low defect density and a uniform single-layer graphene coverage, in agreement with previous results on this growth method [4]. As shown in Fig. 3a, the carbon peak at 550 °C decreases considerably, indicating the formation of volatile carbonaceous species that are desorbed under UHV conditions. The signal remaining for this temperature, located at 284.4 eV, shows an upshift in BE, suggesting the presence of different carbonaceous species through the etching process.

Fig. 3b presents the XPS O 1s spectra of the graphene/Cu foils system in oxygen atmosphere (20×10^3^ L) for different temperatures. The curve fit, made by using Voigt profiles, found two components located at 529.8 eV and 530.7 eV. According to the literature, the main peak, at lower BE, corresponds to the contribution of an atomic phase state, i.e., a low-bound state [52,53]. This component increases strongly in intensity as the sample temperature increases. On the other hand, the small peak at higher BE suggests the presence of Cu_2_O [54]. The absence of new peaks for the C 1s and Cu 2p signals for high temperatures confirms the presence of this atomic phase contribution. The spectral changes at 550 °C, associated with the etching process, show shifts in both components towards higher BE. This is a consequence of the change in the chemical state of the oxygen at the surface during the etching process occurring around 550 °C. The etching mechanism probably leads to the formation of some sub-oxides, which are different from the species present under the graphene layer for lower temperature values. On the other hand, the small component at high BE is visible throughout the process, suggesting that it is not possible, even in this controlled environment, to completely remove a minor contribution of copper oxide on polycrystalline surfaces. Finally, the optimal temperature for oxygen intercalation has been determined to be 500 °C.

**Fig. 4:**
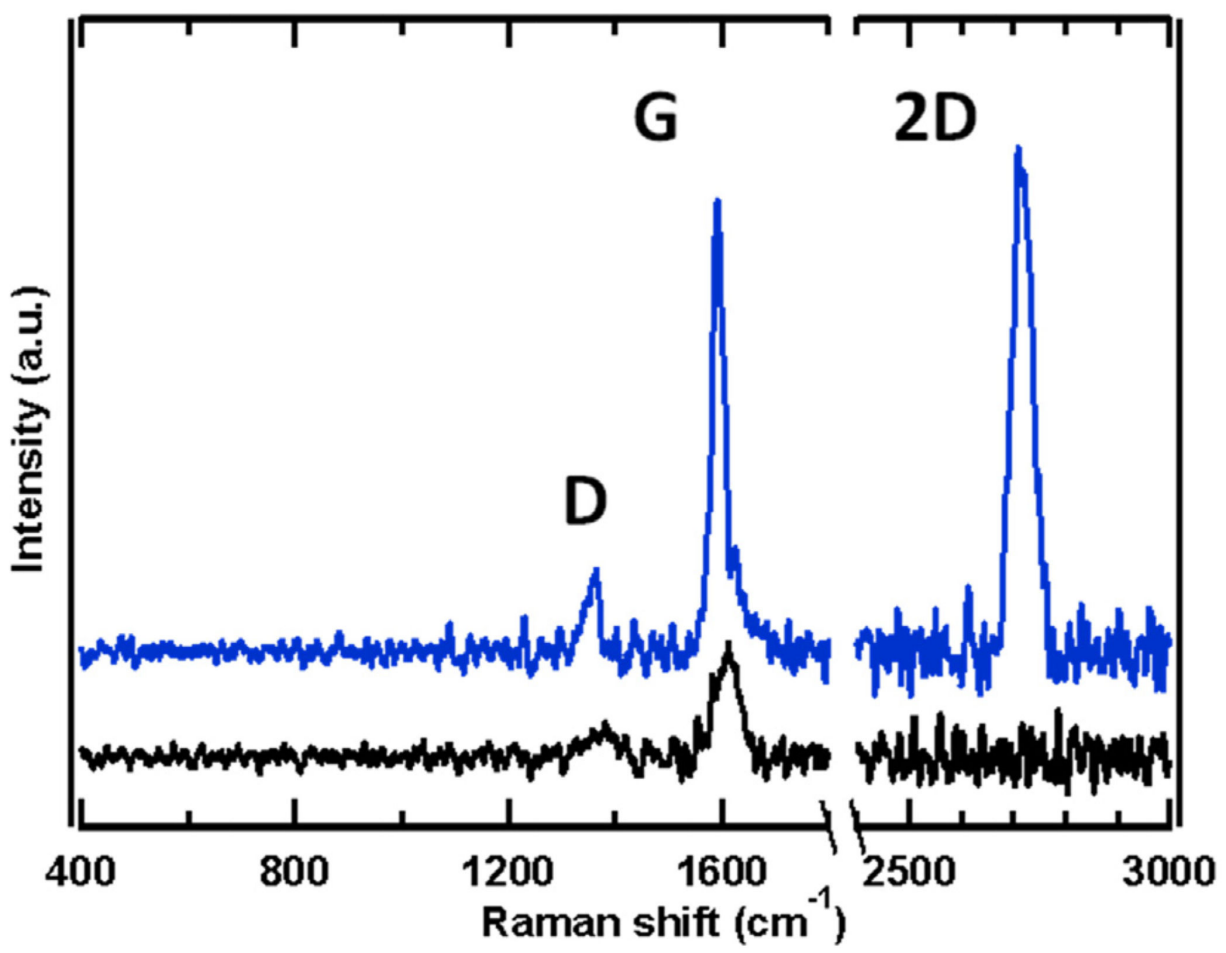
Raman spectra of graphene on polycrystalline Cu foil: as-grown sample (black) and oxygen-intercalated sample (blue).

With the aim of determining the influence of oxygen intercalation on the interaction between graphene and the substrate, Raman spectroscopy was performed ex-situ. Fig. 4 shows two representative Raman spectra corresponding to the as-grown graphene sample on Cu foil (black spectrum) and to the sample after oxygen intercalation (blue spectrum). The Raman spectra were measured at different locations on the Cu foil surface, regardless of the grain orientation. In the spectrum of the as-grown sample the characteristic graphene D and G bands can be seen, but are very weak and notably in all cases the 2D peak is not observed. Weak Raman spectra have been previously reported for graphene on several metal substrates [15,55,56], which has been explained by a quenching phenomenon due to the interaction of graphene with the substrate whose extent has been related to the degree of interaction of graphene with the metal surface, with almost complete suppression of the graphene spectrum when hybridization between metal orbitals and the graphene Dirac cone states is strong [55]. In this respect, the disappearance of the 2D band due to very strong interactions has also been reported for some metal substrates, such as Pt [15,55], Ti [57] and Ni(111) and Co(0001) [58], which has been interpreted as a result of the complete suppression of the Dirac cone near the Fermi level leading to the elimination of the resonant conditions for the second order Raman scattering process [59]. The disappearance of the 2D band could confirm that the PVD approach generates a graphene layer that is more strongly bonded to the Cu surface. On the other hand, in the oxygen intercalated sample (blue spectrum), the 2D peak emerges along with a notable enhancement in the G band, and there is an important downshift of the D and G modes (see Supplementary Fig. S5) that has been associated with relaxation of strain in the graphene sheet, as discussed below.

The 2D band frequency (ω_2D_) has been correlated with strain in graphene layers. However, its absence in the spectra corresponding to the as-grown sample prevents its comparison with the O-intercalated sample by the use of ω_2_D vs ω_G_ vectors, often employed to evaluate both strain and charge density in graphene layers [60,61]. After oxygen intercalation, ω_2D_ appears at an average value of 2718 cm^-1^, corresponding to a blue-shift of > 28 cm^-1^ with respect to free-standing graphene [62]. Previous reports of large blue-shifts in ω_2D_, between 15 and 40 cm^-1^, in CVD-grown graphene on Cu foil [55,63,64] and on single crystal Cu(100) and Cu(111) [20,65] have been attributed mainly to compressive strain. Since every band in the Raman spectrum is affected by strain [61], the average ω_D_ and ω_G_ values of 1373 and 1608 cm^-1^, respectively observed in the as-grown sample, also represent a significant blue-shift with respect to free-standing graphene and are coherent with compressive strain. Regarding the G band, in the as-grown sample a broad band profile is observed with ω_G_ ~ 1608 cm^-1^, albeit with some contribution due to the D’ band. Whereas, in the case of the oxygen intercalated sample, the G-band is much stronger and shifted to a lower average frequency of ω_G_ ~ 1600 cm^-1^, although more asymmetric with a small shoulder corresponding to the D’ peak at around 1620 cm^-1^, as previously reported [66], towards values closer to those of free-standing graphene [62]. Albeit the G peak frequency is nearer to that of an unperturbed graphene layer, the shifted 2D requires rationalization. It is important to note that various studies have suggested that the values of ω_G_ and ω_2D_ cannot be explained solely by charge doping and strain [67], and that variations in the Fermi velocity can alter the *ω_2_D* without affecting the ω_G_ [61,68]. Finally, previous studies on the oxidation of graphene on Cu surfaces have attributed red-shifts in ω_D_, ω_G_ and *ω_2_D* to strain release after oxidation, as mentioned previously, which augment with the growth of the oxide underlayer [20,48] producing corrugation and wrinkling in the graphene sheet [69]. In all these cases, characteristic Raman bands of copper oxides were clearly observed, where the strongest bands expected for Cu_2_O at 148, 219 and 635 cm^-1^, and CuO at 273, 327 and 619 cm^-1^ [70,71]. The Raman spectra showed no evidence for the presence of Cu oxides in the samples prior to or after O-intercalation. This result corroborates the XPS analysis, where almost negligible amounts of Cu_2_O were found for the 400 °C sample, the oxygen atomic phase state being the main O 1 s contribution. Thus, the main conclusions from the Raman data are that the as-grown PVD graphene is highly strained and strongly interacts with the Cu surface and after oxygen intercalation the graphene layer exhibits characteristics closer to that of free-standing graphene, although retaining a certain level of strain according to the *ω_2_D* values observed, which evidences partial electronic decoupling of the graphene layer from the metal. The data obtained from KPFS measurements (Fig. S4) provide further support.

**Fig. 5:**
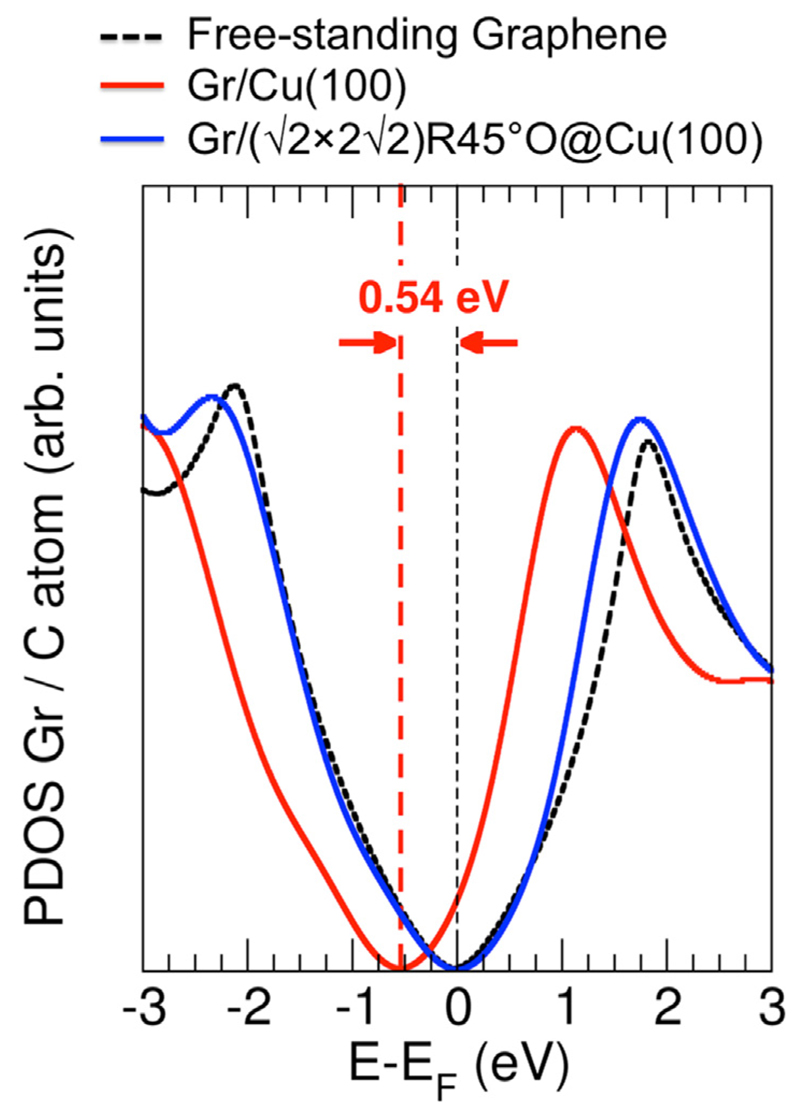
PDOS on the graphene layer for the cases of Gr/Cu(1 00)-(12 × 4)-MR (solid-red line) and Gr/($\sqrt{2}$ × $\sqrt{2}$)*R*45°O@Cu(100)-(12 × 4)-MR (solid-blue line) interfaces as a function of the energy referred to the Fermi energy (in eV). The canonical DOS of a freestanding single layer graphene is also shown (dashed-black line) for a better comparison.

In order to endorse the un-doping process that occurs when oxygen intercalates in the graphene/Cu interface, and according to the experimental evidence, we have carried out (DFT)-based calculations of a clean Cu(100) with the missing row reconstruction, named as Cu (100)-(12 × 4)-MR surface (MR meaning “missing row” structure), of Gr/Cu(100)-(12 × 4)-MR and of Gr/($\sqrt{2}$ × 2$\sqrt{2}$)*R*45°O@Cu(100)-(12 × 4)-MR interfaces; a detailed description of these simulations is found in the supplementary information. The most important result is represented in Fig. 5, which shows the projected density of states (PDOS) per C atom onto the graphene layer for free-standing graphene (dashed-black line), for the Gr/Cu(100) interface (with the Cu surface having a missing row structure) in its most stable configuration (solid-red line) and for the Gr/($\sqrt{2}$ × 2$\sqrt{2}$)*R*45°O@Cu(100)-(12 × 4)-MR interface (with a 0.75 ML of intercalated oxygen in between the Gr/Cu interface (blue-solid line)) as a function of the energy referred to the Fermi energy (in eV). The PDOS profile for the case of Gr/Cu(100) reproduces a well-formed canonical Dirac cone. Nevertheless, the profile morphology shows a slight asymmetry w.r.t. the center of the Dirac cone from that obtained for free-standing graphene (dashed-black line). The symmetry obtained for the free-standing layer is lost when the substrate is considered, due to several factors: firstly, the non-negligible graphene corrugation arising in this case (around 0.17 Å), with regions where the pristine sp^2^ hybridization is slightly broken; secondly, the effect introduced in the electronic properties by the cell strain of around a 2% in the graphene; and finally, the non-negligible chemical interaction arising at discrete points in the interface. Importantly, besides the emerging asymmetry, the PDOS profile for the Gr/Cu(100) case exhibits a significant n-doping level developed in the graphene layer due to the charge transferred from the substrate, in excellent agreement with our experimental evidence and literature [72]. From the calculations, the shift of the Fermi energy between the free-standing graphene and the n-doped case for the Gr/Cu(1 0 0) interface is of around 0.54 eV. This value is of the order of the aforementioned experimental result of 0.77 eV [4]. Interestingly, by inspecting the PDOS profile corresponding to the case of Gr/O@Cu(100) interface, it can be clearly appreciated that the Dirac recovers almost completely the symmetrical distribution of the density of states around the Fermi level, indicating that the n-doping existing in graphene for the case of Gr/Cu(1 00) has completely vanished. This effect may be interpreted as the graphene, with the 0.75 ML of O content underlying, decouples both structurally (see supplementary information) and electronically from the substrate showing a very good agreement with the PDOS profile corresponding to a canonical free-standing graphene layer (dashed-black line).

## 4 Conclusions

In this work, efficient oxygen intercalation between graphene layers grown on Cu by PVD in UHV conditions was reported. The graphene layer preserves its morphology after intercalation. The experimental results evidence the adsorption of an atomic oxygen adlayer on top of the Cu surface with no substrate oxidation. The combination of Raman spectroscopy with the XPS measurements and the KPFS experiments suggest an electronic un-doping of the layer, corroborating the electronic decoupling after intercalation. Theoretical DFT studies show that the system is essentially decoupled before a complete monolayer of oxygen is reached. Therefore, the intercalation method employed was demonstrated efficient to promote electronic decoupling of the graphene layer while retaining the advantages of the growth method. Furthermore, as this decoupling route can be performed immediately after graphene growth, it has the advantage of being an end-to-end process in the same vacuum chamber.

## Supplementary Material

Supplementary data to this article can be found online at https://doi.org/10.1016/j.apsusc.2020.147100.

Supplementary information
