# Supplementary material for: Oxygen intercalation in PVD graphene grown on copper substrates: A decoupling approach
Source: Appl Surf Sci. Author manuscript; Available in PMC 2020 Nov 4. (PMC7116314; doi:10.1016/j.apsusc.2020.147100)
Supplement: Supplementary information [file EMS99318-supplement-Supplementary_information.pdf]

# - Supplementary information -

Oxygen intercalation in PVD graphene grown on copper substrates: a decoupling approach

J. Azpeitia[1], I. Palacio[1], J. I. Martínez[1], I. Muñoz-Ochando[2], K. Lauwaet[1],

F. J. Mompean[1], G. Ellis[2], M. García-Hernández[1], J. A. Martín-Gago[1],

C. Munuera[1], M. F. López[1]

Table of Contents

1. LEED patterns
2. XPS
3. AFM measurements
4. DFT calculations

1. LEED patterns

Figure S1 shows the LEED patterns corresponding to the growth of graphene on the Cu(111) surface. In Figure S1a the pattern of the bare copper surface shows the hexagonal symmetry. The polycrystalline nature of the graphene layer grown on the Cu(111) surface can be observed in figure S1b where a ring (corresponding to the signal of graphene) appears together with the spots of the Cu(111) surface. There is no identifiable surface reconstruction in the pattern corresponding to the oxygen-intercalated sample, shown in figure S1c. The spot corresponding to the copper surface is no longer visible, which can be taken as an indirect evidence of oxygen intercalation.

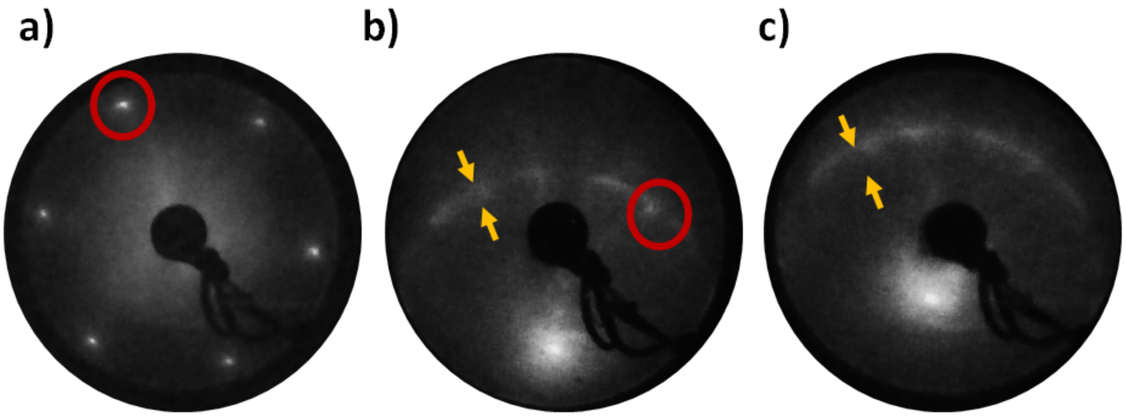

Figure S1. LEED patterns with electron energy of 70 eV of a) bare Cu(111) surface, b) graphene on Cu(111) surface and c) oxygen-intercalated graphene on Cu (111).

2. XPS

Figure S2 shows the XPS Cu 2p spectra obtained during the oxygen intercalation process at different temperatures. No differences in the copper signal are detected on increasing the intercalation temperature. As previously mentioned, the oxygen dose (20x10^3 L) was the same for all temperatures. Since the spectral shapes as well as the peak positions present no changes upon annealing in the oxygen atmosphere, this suggests that the intercalated oxygen covers the Cu surface without generating regions of copper oxide, which would contribute to the signal of the Cu 2p doublet.

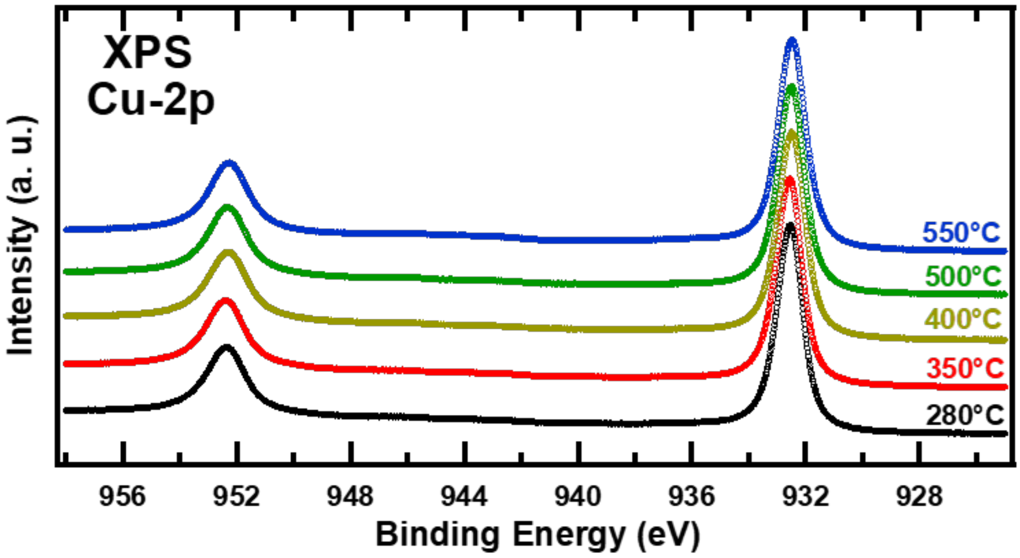

Figure S2. Evolution of the Cu 2p XPS core level spectra of the graphene on polycrystalline Cu foil with the temperature after oxygen exposition at 20x10^3 L.

3. AFM measurements

For the case of Cu (111), an AFM image of the surface prior to growth is shown in figure S3a. The figure shows the surface of the bare Cu, where large terraces with irregular borders can be seen. Some residual contamination is observed in this image as well as in the next one, figure S3b, that exhibits an incomplete graphene layer grown on the Cu(111) sample. In fact, the contamination points appear to act as nucleation points for the graphene growth, as can be seen in figure S3b. A change in the morphology of the Cu surface after growth can be inferred by observing the region underneath the graphene islands, where smaller terraces can be observed. Figure S3c shows an AFM image of a complete monolayer of graphene on Cu (111). Finally, the surface of the oxygen intercalated G/Cu sample as measured by AFM is shown in figure S3d. The Cu terraces

are not visible, suggesting an efficient oxygen intercalation. The presence of the wrinkles is an indirect indication that the graphene layer has not been altered.

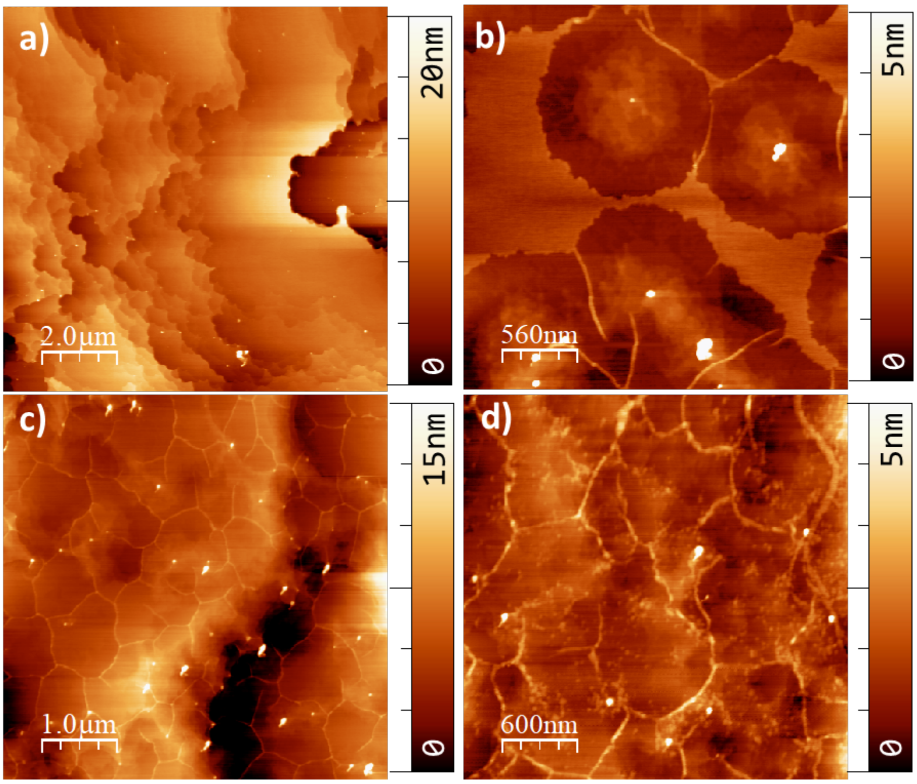

Figure S3. AFM topographic images acquired in dynamic mode showing: a) the Cu (111) surface prior to growth, b) an intermediate stage of the graphene growth where graphene islands partially covering the substrate can be seen, c) the complete graphene layer where the wrinkles cover the whole surface and d) the G/Cu(111) system after the oxygen intercalation process.

The change in graphene doping upon oxygen intercalation observed by XPS and Raman spectroscopy techniques is also corroborated by surface potential measurements with Kelvin Probe Force Spectroscopy (KPFS). Considering the relation between the surface potential (V_{SP}) and the work functions of tip and sample, the following expression is used to relate both magnitudes [1]:

$$eV_{SP} = \Phi_{tip} - \Phi_{sample} \quad (1)$$

Where Φ_{tip} is the work function of the tip, Φ_{sample} is the work function of the sample and e is the charge of the electron. Therefore, changes in the surface potential for different

sample doping is reflected by changes in $\boldsymbol{\Phi}_{sample}$. Thus, by comparing the V_{SP} measured on the as-grown and oxygen-intercalated samples, we can infer the doping trend after the procedure. The critical point in these studies was to guarantee that the tip conditions (particularly, the work function) did not change. To preserve tip conditions, these comparative studies were performed using Kelvin probe force spectroscopy (KPFS) instead imaging acquisition [2,3]. KPFS consists of applying a varying DC bias, to the tip, located above a single spatial location, while monitoring the dynamic response of the cantilever [3,4]. To monitor this, a reference sample of known work function (HOPG in our case, with a value of $\Phi = 4.6\ eV$) was measured between data acquisitions on each of the studied samples The frequency-voltage curves obtained have parabolic voltage dependence, and the position of the minimum yields the V_{SP} value. Representative curves are shown in Figure S4a, for as grown, oxygen-intercalated samples and the HOPG reference.

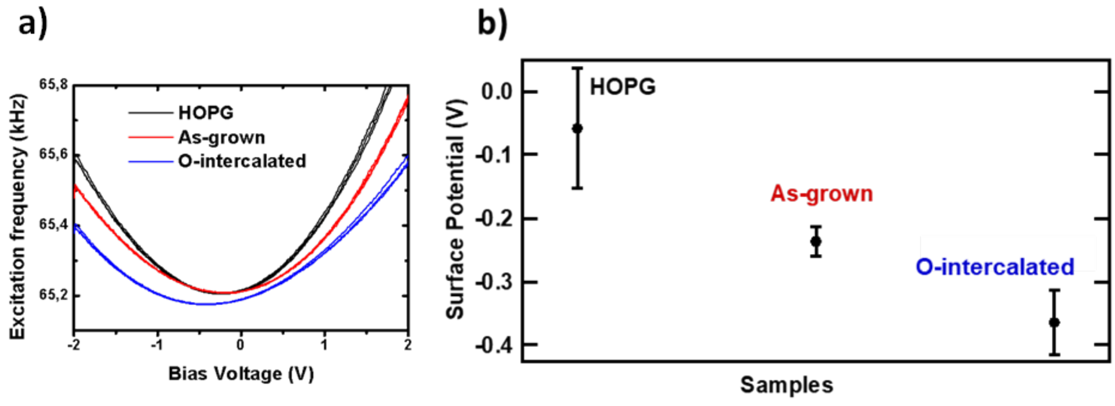

Figure S4. a) Frequency vs. voltage curves acquired on the reference (HOPG), as-grown and oxygen intercalated samples. b) Surface potential measurements of the oxygen intercalated sample, compared to the as-grown sample.

Several curves (thirty three) were acquired from different locations, regardless of the grain orientation, and measurements were repeated altering the sample order, to ensure reproducibility of the measured differences in surface potential. From each curve, a value of the surface potential was obtained. Figure S4b shows the average value of the bias DC voltage of the minimum of the curves obtained, and the standard deviation was plotted as the error bar. The error bars of the two samples (as-grown and oxygen-intercalated) are considerably smaller than the HOPG, demonstrating good-homogeneity of the samples.

A difference in the surface potential of -0.13 V between the as-grown and the oxygen-intercalated samples ($V_{As\text{-}grown}$-$V_{O\text{-}intercalated}$) implies a variations in the work function (equation 1). For our experimental set-up (voltages applied to the tip and samples grounded), a lower surface potential indicates a higher work function value. Therefore, the results presented in Figure S4b indicate that the oxygen intercalated sample has a higher work function than its as-grown counterpart, verifying the hole type doping trend observed in XPS and supported by the behavior observed in the Raman data. Hence, since the as-grown samples have an intrinsic n-type doping, these results provide evidence that the intercalation process diminishes this n-doping character. For this reason, the results reinforce the idea that the oxygen layer acts as an electronic decoupling agent, which would suggest an improved scenario for a transfer process to any desired substrate.

4. Raman measurements

The frequencies of the D, G and 2D bands in the Raman spectra, measured *ex situ* prior to and after oxygen intercalation, were obtained after baseline correction to remove the photoluminescent background due to Cu that is strong at this laser energy (2.1 eV) [5]. Lorentzian bandshapes were employed using the Renishaw WiRE 5.0 curve fitting module in the instrumental software, and the average peak positions for the D and G band frequencies, ω_D and ω_G are represented for both samples in Figure S5, where a significant red-shift is seen on oxygen intercalation. The red dotted lines correspond to the approximate values for ω_D and ω_G in an unperturbed free-standing graphene sheet [6].

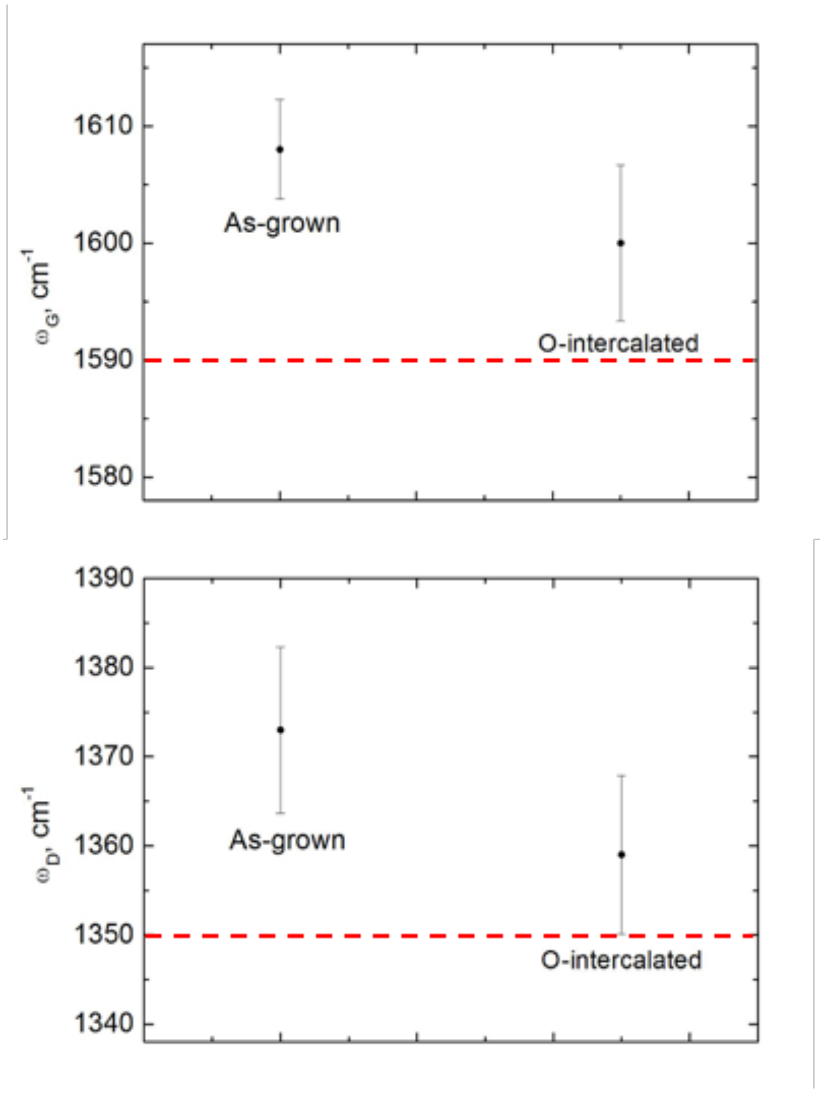

Figure S5. Raman ω_D and ω_G prior to and after oxygen intercalation.

5. DFT calculations

In order to understand the structural and electronic decoupling process of graphene from the copper substrate with an intercalated 0.75 ML oxygen content in between the Gr/Cu(100) interface, DFT based calculations for a clean Cu(100)-(12×4)-MR surface (MR meaning "missing row" structure), for Gr/Cu(100)-(12×4)-MR and for Gr/$(\sqrt{2}\times 2\sqrt{2})R45°$O@Cu(100)-(12×4)-MR interfaces were performed on the basis of the experimental evidence.

Top panel of Figure S6 shows the missing row reconstruction of a Cu(100) surface, highlighting the (12×4) rectangular unit cell used in the calculations (yellow spheres correspond to the topmost Cu atoms, and tan spheres to the rest of Cu atoms, which

permits to appreciate the missing row reconstruction). This unit cell has been adopted for our full cell-structure relaxations with the purpose of accommodating a portion of commensurate-with-the-metal-substrate graphene yielding a strain below 2% after the structural optimization (see below). Four physical Cu(100) metal layers have been considered in our simulations for our metal surface slab (fixing the two bottommost layers during the structural optimization process), as well as periodic conditions to mimic an infinitely extended surface (leaving a minimum distance between neighboring cells along z axis of 15 Å). Once optimized the clean Cu surface, Cu—Cu shortest distances range between 2.57—2.60 Å, with an excellent agreement with the experimental one. Middle panel of Figure S6 shows the structural model including the oxygen atoms (red spheres) of the $(\sqrt{2} \times 2\sqrt{2})R45°$ missing row reconstruction (Cu(100)—O surface), again highlighting the same unit cell used in the simulations than in the previous case with the characteristic rows of alternating O and Cu atoms (O—Cu—O rows) that are separated either by a row of Cu atoms or by a row of vacant sites referred to as "missing row". This arrangement will lead to two distinct types of copper atoms: Cu sites within filled rows, and Cu sites within the O—Cu—O rows. An exhaustive experimental and first-principles STM characterization of this reconstruction can be found in Ref. [7] and references therein. Bottom panel of Figure S6 shows the previously introduced Cu(100)—O surface with an infinite graphene sheet above (dark grey spheres representing C atoms) commensurate with the substrate.

Results of the simultaneous cell-structure relaxation process yield shortest C—C distances in graphene ranging between 1.45—1.47 Å, and a cell strain of around 2% w.r.t. free-standing graphene. After structural optimization graphene locates at around 3.75 Å above the Cu(100)—O surface, to be compared with the 3.25 Å obtained for the case of Gr/Cu(100)-(12×4)-MR interface; which justifies the structural graphene decoupling after the inclusion of the intercalated $(\sqrt{2} \times 2\sqrt{2})R45°$O. Besides, the graphene corrugation (perpendicular distance between the topmost and bottommost C atoms in the graphene layer) is 0.12 Å for the Gr/Cu(100)-(12×4)-MR, which reduces up to < 0.02 Å when considering the Cu(100)—O surface, also reinforcing the idea of an emerging structural undoing.

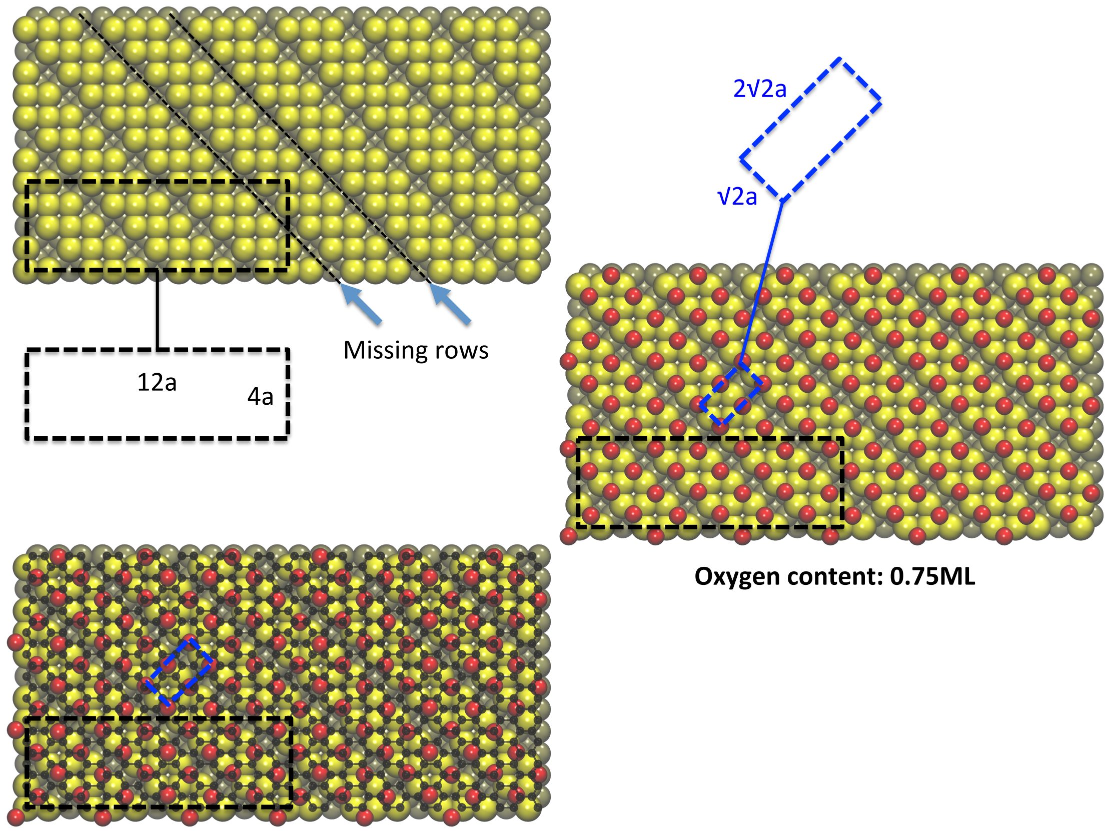

Figure S6. (Top panel) Structural model of the missing row reconstruction of a Cu(100) surface, highlighting the (12×4) rectangular unit cell used in the calculations (yellow spheres correspond to the topmost Cu atoms, and tan spheres to the rest of Cu atoms, which permits to appreciate the missing row reconstruction); (Middle panel) Structural model including the oxygen atoms (red spheres) of the $(\sqrt{2} \times 2\sqrt{2})R45°$ missing row reconstruction (Cu(100)—O surface); (Bottom panel) Structural model of the previously introduced Cu(100)—O surface with an infinite graphene sheet above (grey spheres representing C atoms) commensurate with the substrate.

Besides the explanation in the main text related to the modification of the electronic PDOS on graphene profiles, the electronic charge transferred from the substrate, obtained within the Bader framework [8] decreases from 0.015 up to 0.001 e^{-}/C for the Gr/Cu(100) and Gr/O@Cu(100) cases, respectively, which also justifies a high degree of electronic decoupling after the inclusion of the intercalated O content.

References

[1] M. Nonnenmacher, M.P. O'Boyle, H.K. Wickramasinghe, Kelvin probe force microscopy, Appl. Phys. Lett. 58 (1991) 2921–2923. doi:10.1063/1.105227.
[2] F. Mohn, L. Gross, N. Moll, G. Meyer, Imaging the charge distribution within a single molecule, Nat. Nanotechnol. 7 (2012) 227–231. doi:10.1038/nnano.2012.20.
[3] L. Collins, A. Belianinov, S. Somnath, N. Balke, S. V. Kalinin, S. Jesse, Full data acquisition in Kelvin Probe Force Microscopy: Mapping dynamic electric phenomena in real space, Sci. Rep. 6 (2016) 1–11. doi:10.1038/srep30557.
[4] E. Palacios-Lidón, J. Abellán, J. Colchero, C. Munuera, C. Ocal, Quantitative electrostatic force microscopy on heterogeneous nanoscale samples, Appl. Phys. Lett. 87 (2005) 1–3. doi:10.1063/1.2099527.
[5] A. Mooradian, Photoluminescence of Metals. Phys. Rev. Lett. 22 (1969) 185-187.
[6] T. M. G. Mohiuddin, A. Lombardo, R. R. Nair, A. Bonetti, G. Savini, R. Jalil, N. Bonini, D. M. Basko, C. Galiotis, N. Marzari, K. S. Novoselov, A. K. Geim, and A. C. Ferrari, Uniaxial strain in graphene by Raman spectroscopy: G peak splitting, Grüneisen parameters, and sample orientation, Phys. Rev. B 79 (2009) 205433.
[7] H. Mönig, M. Todorovic, M.Z. Baykara, T.C. Schwendemann, L. Rodrigo, E.I. Altman, R. Pérez, U.D. Schwarz, Understanding Scanning Tunneling Microscopy Contrast Mechanisms of Metal Oxides: A case Study, ACS Nano 7 (2013) 10233.
[8] R.F.W. Bader, Atoms in molecules, Encycl. Comput. Chem. 1 (2002).
